# Unusual cause of a lung mass

**DOI:** 10.4103/1817-1737.30362

**Published:** 2007

**Authors:** Jamal Al Deen Alkoteesh, Amer Shammas

**Affiliations:** *Medical Imaging Department, King Abdulaziz Medical City, Riyadh, Saudi Arabia*

A 29-year-old female presented with complaints of cough, shortness of breath and sputum production for the last few years. There was a history of one episode of hemoptysis. She had no history of fever, weight loss or loss of appetite.

**Clinical examination was unremarkable.**

Chest radiographs [Figures 1 and 2] were obtained followed by a CT thorax [Figures 3 and 4].

**Figure 1:**
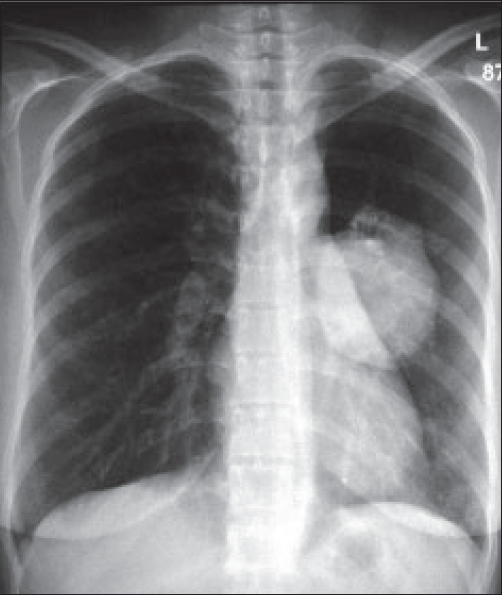
CXR

**Figure 2:**
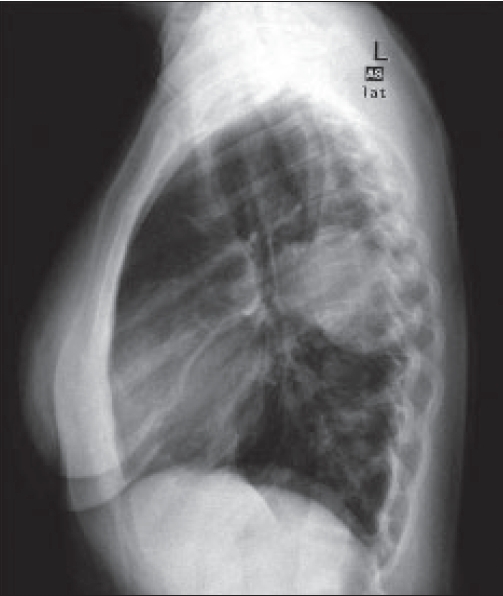
CXR lateral view

**Figure 3:**
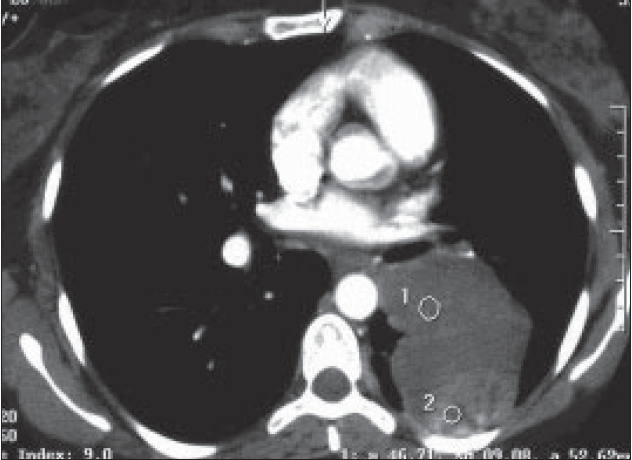
CT 1

**Figure 4:**
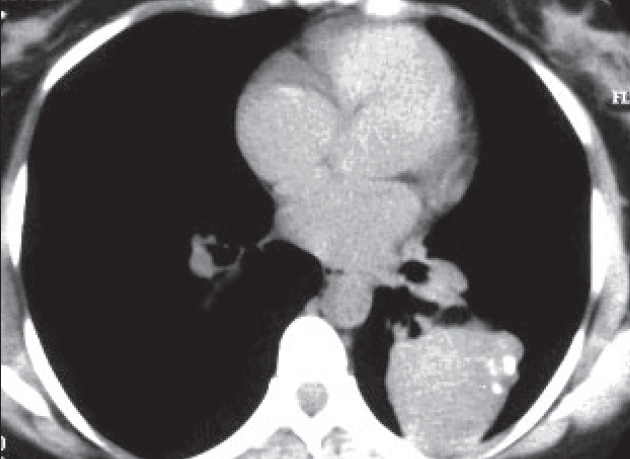
CT 4

## Questions

1. What are the radiological findings on these images?
2. What is the most likely diagnosis and the differential diagnosis of this appearance?

### Radiology findings

On the CT scan, AP and lateral chest X-rays, about 9 cm in diameter oval-shaped solid mass with well-defined borders is seen, and it contains three flicks of calcification. The mass is situated in the left middle lung zone, extending medially into the left lung hilum. Posteriorly, the mass is found abutting the posterior chest wall without causing rib destruction; however, the adjacent rib has slightly increased density. The mass has homogenous density with minimal contrast enhancement. The rest of the lungs were clear, and no mediastinal lymphadenopathy was seen.

### The differential diagnosis

Benign: Lung sequestration, bronchogenic cyst (CXR), hydatid cyst (CXR), chondroma, aspergiloma, neurofibroma, etc.

Malignant: Ewing's sarcoma, PNET, chondrosarcoma, neurofibrosarcoma, malignant fibrous histocytoma, lymphoma, etc.

**Diagnosis: Thoracic Ewing's Sarcoma**

## Discussion

Ewing's sarcoma is the most common primary skeletal tumor of the thoracic cage.[1] Fifteen percent of all Ewing's sarcomas arise primarily in the chest wall, usually from a rib or less frequently from the scapula. It has male predominance of 1.6:1 and typically occurs in children and young adults.

The radiology finding in the chest is a chest wall mass with rib destruction (82%), but mixed lytic-sclerotic (9%) and even sclerotic only (9%). However, Ewing's sarcoma occasionally manifests as a large intrathoracic mass with only a small component of bony involvement.

Extra-osseous intrathoracic Ewing's sarcoma is very rare, and it is usually seen in young adults and manifests as a well-circumscribed, noncalcified mass, more frequently in the paravertebral location. This tumor has propensity to spread inwards towards the lung hilum and into the spinal canal via the intervertebral foraramina.[2]

Involvement of bone marrow, the hallmark of an osseous origin, is typically absent at CT and MRI imaging in these extra-osseous tumors.

One-quarter to one-third of the patients with Ewing's sarcoma have metastases at the time of diagnosis, especially those with tumor close to the trunk. The most common sites of spread are lungs and bones; hence chest CT scan, MRI and bone scan are important in the patient work-up.[3]

Treatment is initially chemotherapy and usually followed by resection with or without radiotherapy. The overall 5-year survival rate is 50%.

## Conclusion

Ewing's sarcoma (extra-osseous or rib origin) should be considered in the differential diagnosis of any young patient who presents with a solid intrathoracic mass.
